# Laparoscopy in the management of stone disease of urinary tract

**DOI:** 10.4103/0972-9941.19264

**Published:** 2005-10

**Authors:** Rajiv Yadav, Rajeev Kumar, Ashok K. Hemal

**Affiliations:** Department Of Urology, All India Institute Of Medical Sciences, New Delhi-110029

**Keywords:** urolithiasis, pyelolithotomy, ureterolithotomy, laparoscopy

## Abstract

As in other fields of urology, the use of minimally invasive techniques has helped decrease the morbidity and convalescence associated with the management of urolithiasis. Laparoscopy has also been used as one of the minimally invasive techniques. This has developed particularly with the increasing experience and use of intracorporeal suturing techniques. However, in comparison with other surgeries, laparoscopy for stone removal is relatively uncommon and we review the current indications, technical limitation and results.

## INTRODUCTION

The surgical management of urinary stone disease has evolved from primarily an open surgical approach to include various minimally invasive options. Increasing experience with shock wave lithotripsy (SWL), ureteroscopy and percutaneous stone removal have markedly decreased the morbidity of treating urinary calculi in conjunction with maximal clearance of stone. Nevertheless, patients may still require open stone surgery, including those in whom SWL or endourologic methods fail or who need simultaneous reconstructive treatment of other urinary tract pathological conditions. These patients, who are otherwise candidates for open surgery, form the principal population that may benefit from laparoscopic surgery in reducing morbidity and hastening recovery. Evolution of technology in recent years has permitted the development of laparoscopy including reconstruction such that it may now be considered for these conditions.[1,2,3,4] Since the indications and techniques of managing renal and ureteral stones are different, laparoscopic management of these two situations will be discussed in this manuscript.

### Laparoscopic pyelolithotomy

Percutaneous nephrolithotomy (PCNL) is the preferred modality for the management of stones more than 3cm in size or for stones with surface area of more than 500mm^24^ Many authors have described the successful laparoscopic management of renal stones though the indications have not been defined and outcomes not compared with PCNL.[5,6] The decision to perform laparoscopic pyelolithotomy or retroperitoneoscopic pyelolithotomy is often arbitrary and depends on the available expertise and equipment. Reconstructing the pyelotomy requires advanced laparoscopic skills for intracorporeal suturing. Goel et al reported a retrospective nonrandomized analysis comparing retroperitoneoscopic pyelolithotomy (RPPL) with PCNL for large renal pelvic calculi and assessed the role of laparoscopic pyelolithotomy in patients of renal stone disease.[7] Apart from demonstrating the feasibility of the procedure, various limitations and difficulties encountered during the procedure were described. A total of 16 cases underwent RPPL with an average stone size of 3.6cm (3.2-4.5) over a period of 7 years. Compared to a similar group of patients that under went PCNL, RPPL took longer operating time (average time 142.2min v/s 71.6min). However, estimated blood loss, hospital stay and mean duration of return to full activity were similar to PCNL. Apart from the initial learning curve, various other reasons were thought to be responsible for longer operating time in RPPL. These included time required for the dissection of renal pelvis, pyelotomy, stone removal and pyelotomy closure by intracorporeal suturing. One of the major limitations of the laparoscopic approach noted by them was the difficulty in retrieving the caliceal calculi. Stone removal required flexible nephroscope or cystoscope through one of the ports. Micali et al., have also described removal of pelvic and caliceal calculi using the flexible cystoscope through the 10/12 mm laparoscopic port.[4] An intrarenal pelvis is difficult to dissect because of limitation of space and difficulty in retracting the renal parenchyma to expose the renal sinus. Moreover, applying intracorporeal sutures in such a pelvis is often difficult. Sometimes, the presence of a stone gives rise to adhesions in the peripelvic area, making dissection difficult and bloody. Conversion to open surgery may be required due to significant perinephric adhesions and resultant difficulty in dissection.[5,7] All these factors are insignificant in PCNL thus making it the treatment of choice in such situations. However, in situations of failed percutaneous access due to technical reasons, a laparoscopic approach in selected cases may provide a similar success rates as open surgery. Casale et al[8] have reported a retrospective analysis of record of 8 such patients with symptomatic non-obstructing renal stones in whom percutaneous access failed and who subsequently underwent transperitoneal laparoscopic pyelolithotomy. The authors preferred the transperitoneal approach in this particular scenario, anticipating the fibrosis caused by multiple failed attempts at percutaneous access making the retroperitoneoscopy difficult. Interestingly the operations were performed in children of age 3 months to 10years (mean age 4 years). Inclusion criteria were failed percutaneous access secondary to a non-dilated system, failed SWL or a stone burden greater than 2.5 cm[2]. Three ports were used for a transperitoneal approach. After pelviotomy, the stones were removed using both rigid and flexible cystoscopes. Stones within the calices were extracted with a nitinol stone basket. In situations where the stone was too large for the port site, it was placed in a laparoscopic sac and removed via the umbilical laparoscopic port site by extending the incision after the pelviotomy was repaired. After closure of the pelviotomy with a running 5-0 absorbable polyglycolic acid suture, indigo carmine was injected into the renal pelvis to confirm a water tight closure. Average operative time was 1.6 hours (range 0.8 to 2.3). Mean estimated blood loss was 15ml (range 0 to 50ml). Mean hospital stay was 2.15 days (range 0.5 to 6). A range of 1 to 3 stones (median 1) were removed and mean stone burden was 2.9cm[2]. An open ended ureteric catheter (4F) was kept for 1 day. At three months, on ultrasonographic evaluation, none of the patients had residual calculi, resulting in a procedural stone free rate of 100%.

Another special situation where laparoscopic pyelolithotomy has been described is pelvic or ectopic kidney with stones. Laparoscopic pyelolithotomy is a good option for the treatment of such stones because pelvic kidneys can be approached laparoscopically with standard pneumoperitoneum.[9] Peripelvic inflammation as well as a number of aberrant vessels may be found while dissecting the pelvis and this requires expertise in laparoscopic dissection.

Thus, although the laparoscopic approach cannot be applied universally in cases of renal stone disease, it may be a beneficial approach in selected situation like failed PCNL and stone in ectopic kidneys.

### Laparoscopic anatrophic nephrolithotomy

For complex kidney stones anatrophic nephrolithotomy (AN) may be a cost effective alternative to multiple endourological treatment sessions.[10] As the laparoscopic approach is gaining increasing importance in present day urologic surgery, Kauok et al[11] determined the technical feasibility of laparoscopic AN in a chronic survival porcine model and studied the surgical outcome and related complications. A large animal model for a staghorn renal calculus was created using polyurethane isopolymer. After developing the laparoscopic technique in 3 acute animals, the chronic survival study was performed in 10 consecutive female pigs weighing 30 to 40 kg each. In the first stage, a staghorn stone was created by retrograde injection of polyurethane mixture through the ureteral catheter into the renal pelvis with simultaneous occlusion of UPJ with a ureteral balloon catheter. Second stage was performed two weeks after the formation of the staghorn calculus. This interval allowed the development of hydronephrosis in the kidney. A 4 port transperitoneal approach was used. En-bloc clamping of the renal artery and vein was achieved using a laparoscopic Satinsky clamp after prior kidney mobilization and dissection of the renal hilum. Surface cooling using intracorporeal ice slush was performed in 1 animal. An EndoCatch II (US Surgical, Norwalk, Connecticut) bag was positioned around the kidney and filled with ice slush introduced through a 12mm port. After clamping the hilum, the kidney was allowed to cool for 10min. For lateral incision of the renal parenchyma and collecting system in 5 animals, intraoperative laparoscopic ultrasonography was used to identify the site of thinnest parenchyma and Doppler to locate an avascular plane through the thinned parenchyma. The stone was retrieved in a 10mm Endocatch bag. Subsequently, laparoscopic ultrasonography was repeated or flexible endoscopy of the caliceal system and upper ureter was performed to identify and retrieve any residual stones to achieve complete stone clearance. The collecting system and renal parenchyma was continuously sutured with no. 2 polyglactin absorbable suture on a GS-21 needle in a watertight manner. The procedure was successful in all 9 unilateral and 1 staged bilateral laparoscopic AN. Total mean operating time was 125 min (range 70–180) and mean blood loss was 68cc. Mean warm ischemia time was 30 minutes (range 23–39), including a mean of 11.5 min for renal parenchymal incision and stone extraction, 9 min for caliceal repair and 8.3 min for renal parenchymal repair. Mean preoperative baseline creatinine level was 1.6mg/dl, while after stone formation and after AN was 1.9mg/dl and 1.8mg/dl, respectively, at a mean 4.4 weeks of follow up. Comparative Tc-diaminetetramine pentaacetic acid (DTPA) renal scan performed before and 1 month after laparoscopic AN demonstrated significant improvement in function in 9 renal units, including 1 case of bilateral AN. The 2 preoperative nonfunctioning hydronephrotic kidneys did not improve after nephrolithotomy. Subsequent autopsy bench angiography documented preservation of the major intrarenal vasculature and histological examination revealed complete healing of the nephrolithotomy incision site.

### Caliceal diverticular stones

The incidence of calculi within caliceal diverticulae has been reported to be between 10% and 50%.[12] Laparoscopy as an option for management of symptomatic caliceal diverticular stones is considered when the other minimally invasive options (i.e. SWL, PCNL and ureterorenoscopy) are not feasible. Laparoscopic treatment is best reserved for cavities located anteriorly (which exclude direct percutaneous access) and without significant overlying parenchyma, cavities that are large or have an endoscopically inaccessible neck (precluding ureterorenoscopy) and those with either a narrow neck or large stone burden (precluding SWL).[13] Various authors have described laparoscopic treatment of a stone-filled caliceal diverticulum; the cortex in such situations is usually thin and no difficulty is encountered localizing the stone with instrument palpation and needle aspiration.[14] However, in situations of thicker overlying cortex, bleeding from the nephrotomy incision may be difficult to control. Unlike a percutaneous procedure, Gerota's fascia is opened and spontaneous compression is unlikely. Laparoscopic ultrasonic imaging and color Doppler ultrasonography may allow an optimal selection of the site of incision closest to the stone at the thinnest location of the cortex, and away from major vessels that are visualized with color Doppler ultrasonography. Cangh *et al*[15] have described the use of laparoscopic ultrasound scanner while doing laparoscopic nephrolithotomy for symptomatic stones located in an obstructed anterior calyx. The authors use an argon beam coagulator while incising the renal cortex as this facilitates hemostasis. Use of an argon beam coagulator has previously been demonstrated in various intracavitary indication, including laparoscopic partial nephrectomy.[16–17] Simultaneous laparoscopic and ultrasonographic guidance helped in stone localization and selection of an optimal site for cortical incision. The risk of residual fragments is also minimized by perioperative ultrasound examination, as even very small fragments are clearly demonstrated.

### Laparoscopic pyelolithotomy with pyeloplasty

When there is concomitant ureteropelvic junction obstruction with stones, the gold standard has been open pyeloplasty and pyelolithotomy with a success rate of 90%.[18] Several endoscopic techniques have been developed as alternatives to open pyeloplasty and pyelolithomy with variable results. Antegrade endopyelotomy with concomitant percutaneous stone removal has been performed with a success rate of 64% to 85%.[19–20] Ureteroscopic endopyelotomy or endoureterotomy with a ureteral balloon device has also been used to treat ureteropelvic junction obstruction with a success rate of 78%.[21] However, in cases of coexisting stones, the obstructed segmented may prevent stone passage after SWL or ureteroscopy. Recent developments have combined the benefits of minimally invasive surgery with the higher success rate of open approaches by developing laparoscopic pyeloplasty. [1,2] While there is increased invasiveness compared with endopyelotomy, laparoscopic pyeloplasy appears to provide a higher success rate than incisional techniques. Compared with traditional approaches the major disadvantages of the laparoscopic pyeloplasty are longer operative time and a steep learning curve.[22] Ramakumar et al[23] have presented their experience with laparoscopic pyeloplasty plus pyelolithotomy in patients in whom stones were not the cause of UPJO. A transperitoneal approach was used in 19 patients (20 renal units). A flexible cystoscopy and C-arm fluoroscopy retrograde pyelography was done to delineate the anatomy of the upper urinary tract and to determine the exact number and location of stone. A ureteral double pigtail stent was placed with the proximal end in the upper calyx and away from the renal pelvis. The authors preferred the transperitoneal technique to maximize the working space and anatomical orientation. Three ports were placed after the creating pneumoperitoneum. All ports were placed in the midline, namely a 10mm trocar in the umbilicus for the camera, a 5mm trocar between the xiphoid and umbilicus, and a 12mm trocar between the umbilicus and symphysis pubis. After the dissection of the renal pelvis and making it free from the adjacent structures, pyelolithotomy was performed before the pyeloplasty. The authors have emphasized the importance of prior decision regarding the technique for pyeloplasty before making the incision to optimize the pyelotomy incision placement. The initial incision should not be too large to prevent avulsion of the UPJ during pyeloscopy. Pelviotomy is eventually incorporated into the final pyeloplasty. Stones were removed using spoon forceps. For stones that could not be removed by rigid instruments, flexible cystoscope was placed through the inferior laparoscopic port and pyeloscopy performed under direct vision. Stones in the calyces were extracted with a triradial grasper or stone basket. Larger stones were either fragmented with the holmium laser or alternatively placed in a specimen retrieval bag and removed at the end of surgery. Smaller stones fragments were flushed out by the cystoscope irrigation fluid and removed from the renal pelvis or retroperitoneum using the laparoscopic irrigator aspirator. After calculi removal, the operation proceeds as standard laparoscopic pyeloplasty. Anderson- Hynes dismembered pyeloplasty was performed in 11 out of 20 units, Y-V plasty in 8 and Heinecke Mickulicz procedure in 1. The authors have described certain technical maneuvers to facilitate the stone removal. It is important not to completely transect the ureter before removal of all the stones and subsequent pyeloplasty. The renal pelvis tends to retract after detachment, thus, impeding passage of the cystoscope and grasping forceps. Maintaining the continuity of the UPJ stabilizes the pyelotomy for stone extraction. The laparoscopic suction device is placed below the renal pelvis to aspirate continuously irrigation fluid form the operative field. To confirm stone position and complete stone removal, intra-operative fluoroscopy is useful. Average operating time in this series was 4.6 hours (range 2.3–6.2), with a mean estimated blood loss of 145ml (range 30-370). Mean hospital stay was 3.4 days (range 2-6). A median of 1 stone (range 1–28) was removed and the mean stone burden was 1.4cm.[2] At three months, 2 patients had residual calculi, resulting in a procedural stone-free rate of 90% (18–20). These patients underwent further stone management, including ureteroscopy or SWL. At a mean radiological follow-up of 11.3 months (range 3–43.3) 18 of the 20 patients (90%) had no evidence of obstruction and/or improved hydronephrosis on IVU (10), diureteric renography (6), computerized tomography (3) or renal ultrasound (1). The techniques that failed in 2 patients were Y-V plasty and Heinecke Mickulicz procedure.

### Laparoscopic assisted percutaneous nephrolithotomy in ectopic kidneys

Management of nephrolithiasis within an ectopic kidney presents a challenge to the urologist. PCNL is still not universally performed for calculi in ectopic kidneys because of fear of injury to abdominal viscera and vessels. Ectopic kidneys require a different and more complicated approach for PCNL. Eshghi *et al*[24] first described a laparoscopic assisted PCNL technique for ectopic kidneys. Percutaneous access was obtained by retrograde nephrostomy puncture in combination with continuous observation and displacement of bowel loops via a laparoscope. This system consisted of a 5F angiographic catheter with a curved tip sheathed in a 9F catheter. The lumen of the smaller catheter admitted a hollow 160cm–20gauge steel needle that punctured the chosen renal calyx and also permitted passage of a firm narrow guide wire. The tip of the 5F catheter was directed into an upper anterior calyx under fluoroscopic control. As the 9F catheter was advanced into the calyx, the needle stylet was pushed through the renal parenchyma under observation thorough the laparoscope. The stylet was engaged with grasping forceps and pulled out of the abdominal cavity via the trocar in the right lower quadrant. The 5F and 9F catheters then were advanced over the stylet to secure the nephrostomy tract, after which the stylet was removed and a second (safety) guide wire was passed through the 9F catheter. The nephrostomy tract was then sequentially dilated to 34F through the abdominal wall with Amplatz dilators with the help of laparoscopic vision and fluoroscopy. A nephroscope was introduced into the renal pelvis through the 34F sheath. Holman et al[25] reported 15 patients treated with laparoscopic assisted percutaneous transperitoneal nephrolithotomy. With the patient in the Trendelenburg position under laparoscopic control, the bowel was dislodged until the kidney became visible, allowing percutaneous access. All stones were removed successfully with minimal morbidity. Zafar *et al*[26] modified the laparoscopic technique to include intracorporeal suturing of the nephrotomy site and ureteral stent placement allowing elimination of a transperitoneal nephrostomy tube. Troxel *et al*[27] described extraperitoneal laparoscopy-assisted percutaneous approach to access the lower-pole calyx of a pelvic kidney for percutaneous nephrolithotomy.

### Laparoscopic management of ureteric calculi

Since the introduction of SWL[28] and ureteroscopy[29] for the management of ureteric calculi, the routine use of an open surgical approach for removal of ureteric calculi has rapidly declined. However, large ureteric calculi pose significant challenge for modern endourologic techniques, often requiring several endoscopic procedures as well as SWL sessions. SWL is found to be suitable for managing ureteric stones of < 1cm.[30] As the stone size increases, the chance of clearance decreases[31] and of the need for multiple sessions increases. Park *et al*[32] reported that the stone free rate decreased from 84% to 42% when the stone is > 1cm. Thus the indications for laparoscopic ureterolithotomy in the age of modern endourology include stones which cannot be accessed ureteroscopically or cannot be fragmented. Various authors would also consider large (e” 15mm) proximal ureteric stones a relative indication.[33,34] Advantage of laparoscopic ureterolithotomy for large upper ureteric stones is the high stone free rate after a single procedure, thereby allowing early return to regular activities. The disadvantages include a longer hospital stay, the risk of injury to intra-abdominal structures inherent in the laparoscopic approach, and the risk of conversion to open surgery. In addition, the risk of postoperative prolonged urine leak or urinoma[34–36] is also reported. Laparoscopic ureterolithotomy may be done transperitoneally[33,36–38] or retroperitoneally.[34,39,40] Distinct advantages of retroperitoneoscopic surgery over transperitoneal access have been described[34] which include avoidance of contamination of the peritoneal cavity, the patient has no shoulder tip pain, previous abdominal surgery does not preclude this approach and there is a lower incidence of long-term complications, such as port site hernia and bowel obstruction. In addition, bowel mobilization is not required thus the risk of inadvertent gut injury an ileus is minimized and retraction of the solid viscera is not required. Transperitoneal approach gives excellent working space and it is easier for a laparoscopist to start with.[41] For transperitoneal approach, patient is placed in the flank position.[33] Laparoscopic ports are placed a) at the lateral edge of the rectus, level with the umbilicus b) in the anterior axillary line below the costal margin and c) in the anterior axillary line in the iliac fossa. A fourth, if needed for assistance or suction, post may be placed in the mid-axillary line, level with the umbilicus. The colon is reflected medially and the ureter is exposed. A vertical incision is made in the ureter over the stone and the stone is removed. For retroperitoneal approach, the patient is placed in the lateral flank position.[42] The bridge of the table is elevated to flatten the lumbar region. Anterior tilt may help to avoid peritoneal transgression during the port placement by causing the contents of peritoneum to fall forward. A 1.5 cm incision is made 2cm below and posterior to the tip of the 12^th^ rib in the posterior axillary line. The incision is deepened down to the thoracolumbar fascia. Layer dissection is to be avoided to prevent surgical emphysema. A retroperitoneal space is created using either a commercially available or an indigenously built gloved finger balloon. Another 2 ports (two, 5mm, a 5mm and a 3mm, or two, 3mm) are inserted under laparoscopic vision to avoid transgression of the peritoneum. Once inside the retroperitoneal space, the ureter is identified anterior to the psoas muscle. While dissecting the ureter, its identity is confirmed by visualizing ureteric peristalsis and the bulge caused by the stone. In situation of accidental peritoneal tear, it is still possible to manage laparoscopically by the following maneuvers: a) placing a fan retractor over the peritoneum b) reducing the peritoneal pressure by placing a Veress needle in the abdomen c) by enlarging the peritoneal tear to equalize pressure or d) by conversion to a transperitoneal approach. Difficulty in locating the stone due to severe peri-ureteric fibrosis can be overcome by the use of fluoroscopy or intraoperative laparoscopic ultrasonography. Stone migration can be prevented by holding the ureter with Babcock forceps proximal to the stone or, if the ureter is dilated, by looping a ribbon or umbilical tape. Levering the stone out of ureter prevents its breakage and subsequent problems with small pieces. A grasper should be used to extract the stone only when the stone is hard, otherwise grasping the stone can break the stone with possibility of migration. Most groups prefer to stent the ureter[37,39,40,43,44] and then close the ureterotomy[40,45] or leave it open.[43,44] No significant differences in the duration or urine leakage from the drain have been reported using any of these techniques. The duration of urine leakage is probably longest when the ureterotomy is neither sutured nor stented. [40,43,44] In a series of 101 laparoscopic ureterolithotomies, Gaur et al[46] neither stented nor sutured the ureter in 48 patients (6 of these had a percutaneous nephrostomy in place). In the remaining 45 cases, the ureter was sutured in 36, stented in 18 and both sutured and stented in 9. The overall mean urinary leakage was 5.5 days: it was 7.1 days when the ureter was left open, 5 days when it was only stented, 4.4days when it was only sutured and 3.2 days when it was both stented and sutured. There was prolonged (> 7 days) leakage of urine in 20 patients. In 14 of these the ureter was neither stented nor sutured, whereas 6 had their ureters sutured. According to the authors, the probable reason for urine leak in the sutured ureters was that these ureters were chronically inflamed and edematous and friable after infection and prolonged impaction. In such circumstances, the ureters should only be stented and not sutured.

**Table 1:** Comparative results of laparoscopic ureterolithotomy

Series	Sinha[40]	Turk[38]	Keeley[33]	Gaur[46]	Hemal[34]
No of procedure	24	21	14	101	31
Access	RP-24	TP-21	TP-14	TP-1	RP-31
Transperitoneal				RP-100	
(TP)/Retroperitoneal(RP)					
Mean stone size (millimeters)	-	-	27	16	22
Mean operative time (min)	61	90	105	79	67
Hospital stay (days)	3.6	-	5.6	3.5	2.4
Success %	100	90	100	92	100
Complications %	-	-	21	-	6.4
early delayed	-	-		4	-

## CONCLUSIONS

Laparoscopic approach should be utilized for stone management in urinary tract where SWL, PCNL and ureteroscopy have failed or deemed unsuitable. It is also a viable option in patients with unusual anatomy such as a pelvic kidney with stone resistant to fragmentation.
